# Aqueous Supercapacitor with Wide‐Temperature Operability and over 100,000 Cycles Enabled by Water‐in‐Salt Electrolyte

**DOI:** 10.1002/cssc.202401681

**Published:** 2024-11-15

**Authors:** Zahid Ali Zafar, Radim Weisser, Ghulam Abbas, Martin Silhavik, Prabhat Kumar, Jiří Červenka

**Affiliations:** ^1^ Department of Thin Films and Nanostructures FZU - Institute of Physics of the Czech Academy of Sciences, Cukrovarnická 10/112 Prague 162 00 Czech Republic; ^2^ IMDEA Materials Institute Getafe 28906, Madrid Spain; ^3^ Faculty of Chemical Engineering University of Chemistry and Technology Prague CZ-166 28, Technická 5, Praha 6 Czech Republic; ^4^ Waterloo Institute for Nanotechnology University of Waterloo Waterloo ON N2 L 3G1 Canada

**Keywords:** Water-in-salt, Aqueous electrolyte, Wide Electrochemical potential window, Double layer capacitor, Supercapacitor, Activated carbon

## Abstract

Supercapacitors are crucial in renewable energy integration, satellite power systems, and rapid power delivery applications for mitigating voltage fluctuations and storing excess energy. Aqueous electrolytes offer a promising solution for low‐cost and safe supercapacitors. However, they still face limitations in cycle life and wide‐temperature range performance. Here, we present a symmetric supercapacitor utilizing activated carbon electrodes and a “water‐in‐salt” electrolyte (WiSE) based on lithium perchlorate. The WiSE electrolyte exhibits an expanded electrochemical stability window, endowing the aqueous supercapacitor with remarkable stability and long cycle life of over 100,000 cycles at 500 mA g^−1^ with more than 91 % capacity retention. Moreover, the supercapacitor demonstrates good rate capability and wide temperature operability ranging from −20 to 80 °C. The use of high concentrations of salt in the aqueous electrolyte contributes not only to the enhancement of supercapacitor performance and cycle life but also to the temperature stability range, enabling all‐season operability.

## Graphical abstract

This study presents a symmetric supercapacitor with activated carbon electrodes and a “water‐in‐salt” electrolyte (WiSE) based on lithium perchlorate, offering over 100,000 cycles with over 90 % capacity retention. The supercapacitor operates efficiently from −20 to 80 °C, enhancing performance, stability, and all‐season operability.

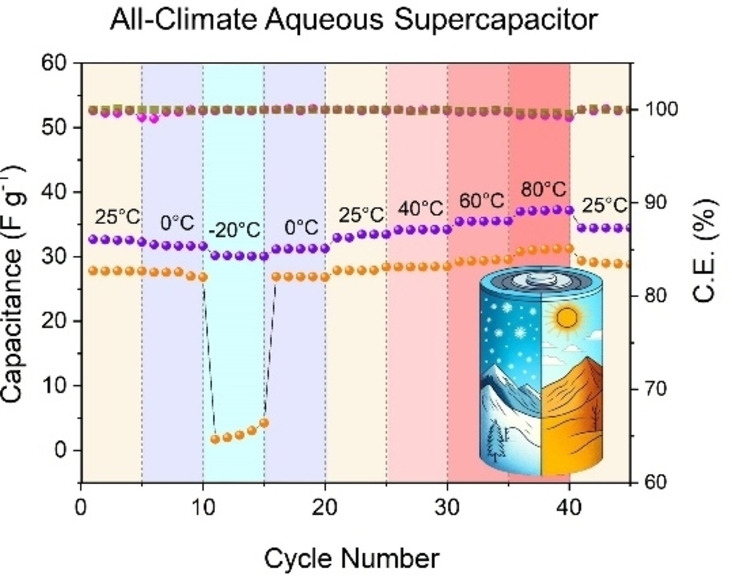

## Introduction

Electrical double‐layer capacitors, commonly known as supercapacitors (SCs), serve as energy storage systems that store energy through the adsorption and desorption of ions at the electrode‐electrolyte interface.^[1]^ SCs play a vital role in renewable energy integration by providing short‐term energy storage and smooth power transmission, mitigating power fluctuations. Due to their low cost, long lifespan, and the ability to quickly deliver high currents, they can be used in various fast power and energy delivery applications.^[2]^ Furthermore, SCs can find applications in the aerospace and defense sectors in satellite systems, smart communications, and navigation.^[3]^ Ideal SCs should operate over a wide working temperature range and demonstrate longevity, non‐flammability, and cost‐effectiveness, all of which heavily rely on the characteristics of the electrolytes employed.^[5]^

Presently, three classes of electrolytes, namely aqueous, organic, and ionic liquids, have been utilized in SCs.^[7]^ Although organic electrolytes are widely used in commercial SCs due to their wide voltage window, their flammability and high cost remain significant drawbacks.^[9]^ Alternative electrolytes, such as ionic liquids, have recently garnered significant attention due to their favorable properties, such as good ionic conductivity, electrochemical stability, low vapor pressure, and reduced flammability. However, ionic liquids face disposal challenges posed by toxicity and high cost, which strongly hinder their broader applications in batteries and SCs.^[12]^ Moreover, the assembly of SCs based on organic and ionic liquid electrolytes necessitates access to controlled water and an oxygen‐free environment, thereby increasing manufacturing difficulties and costs.^[7]^

Aqueous electrolytes would be ideal electrolytes for SCs due to their high ionic conductivity, low cost, non‐flammability, and ease of device fabrication.^[13]^ However, the limited operating potential window of aqueous electrolytes, resulting from the low thermodynamic stability window of water (1.23 V), and the narrow working‐temperature range of water freezing at low temperatures and evaporation at high temperatures, pose significant challenges impeding the development of aqueous electrolyte‐based SCs.^[17]^ Therefore, it is important to enlarge the operating potential window and expand the working‐temperature range of aqueous electrolytes to enhance the performance of aqueous SCs.^[19]^

Recent studies have shown that the addition of high salt concentration can effectively modulate the electrochemical stability window (ESW), as well as the freezing and boiling point of electrolytes.[^13^, ^20^] Highly concentrated “water‐in‐salt” electrolytes (WiSE) with a weight ratio of salt to solvent exceeding 1, first introduced by Suo et al. using a 21 *m* (mol kg^−1^) LiTFSI water system, enlarged the electrochemical stability window (ESW) over 3 V^[22]^. There have also been reported several other WiSE systems for SCs, for instance 12 *m* NaNO_3_ by Yan et al. and 27 *m* KOAc by Soavi et al.,[^7^, ^23^] which showed operating voltages of 2.1 V and 1.8 V, respectively. WiSE electrolyte systems suppress the activity of water molecules, primarily involved in the solvation sheath of metal ions, thereby reducing the presence of free water molecules in the system and enlarging ESW.^[26]^ Further, the right choice of salts in the WiSE systems can also significantly affect the ESW and reduce costs compared to high‐concentration electrolytes containing fluorine, for instance LiTFSI, etc..^[25]^ Recently, our group extensively studied the influence of anion nature (chaotropic/kosmotropic) on water structure and the ESW in different WiSEs.[^9^, ^13^, ^27^] Specifically, chaotropic perchlorate anion was found to be supportive in suppressing water activity and widening the ESW in aluminum‐ and zinc‐based WiSE systems. Another strategy how to further expand the operating window of WiSE involves organic/inorganic hybrid electrolytes by incorporating organic solvents like trimethyl phosphate (TMP), acetonitrile (ACN), etc..[^1^, ^29^] However, WiSE systems composed of highly concentrated organic or inorganic salts present challenges such as high cost, increased viscosity, low conductivity, and low‐temperature salt.[^7^, ^29^] Although the introduction of organics can suppress the freezing of water, it reduces the ionic conductivity of electrolytes.^[20]^ Additionally, adding an organic solvent into WiSE inadvertently weakens the coordination structure between metal cations and water molecules, particularly in high‐temperature environments. Consequently, SCs employing hybrid electrolytes struggle to operate at high temperatures (>60 °C) and high working electrochemical potentials.^[31]^ In the present study, we demonstrate the application of a Li‐perchlorate (LiClO_4_)‐based WiSE for SCs. Specifically, we report a symmetric supercapacitor utilizing activated carbon (AC) as the electrode material in 7.5 *m* (molal) LiClO_4_ WiSE. We combine a high surface area commercial AC with as‐formulated LiClO_4_‐WiSE and compare its performance with a diluted 0.5 *m* LiClO_4_ aqueous electrolyte.

We show that the WiSE demonstrates superior performance in SCs, resulting in exceptional stability and an impressive long‐cycle life of over 100,000 cycles at 500 mA g^−1^. Furthermore, this supercapacitor demonstrates reliable performance over a wide temperature range, from −20 °C to 80 °C. Overall, this study contributes to the advancement of SCs by exploring the potential of aqueous electrolytes with an expanded operating potential window and a widened working‐temperature range.

## Results and Discussion

**Figure 1:**
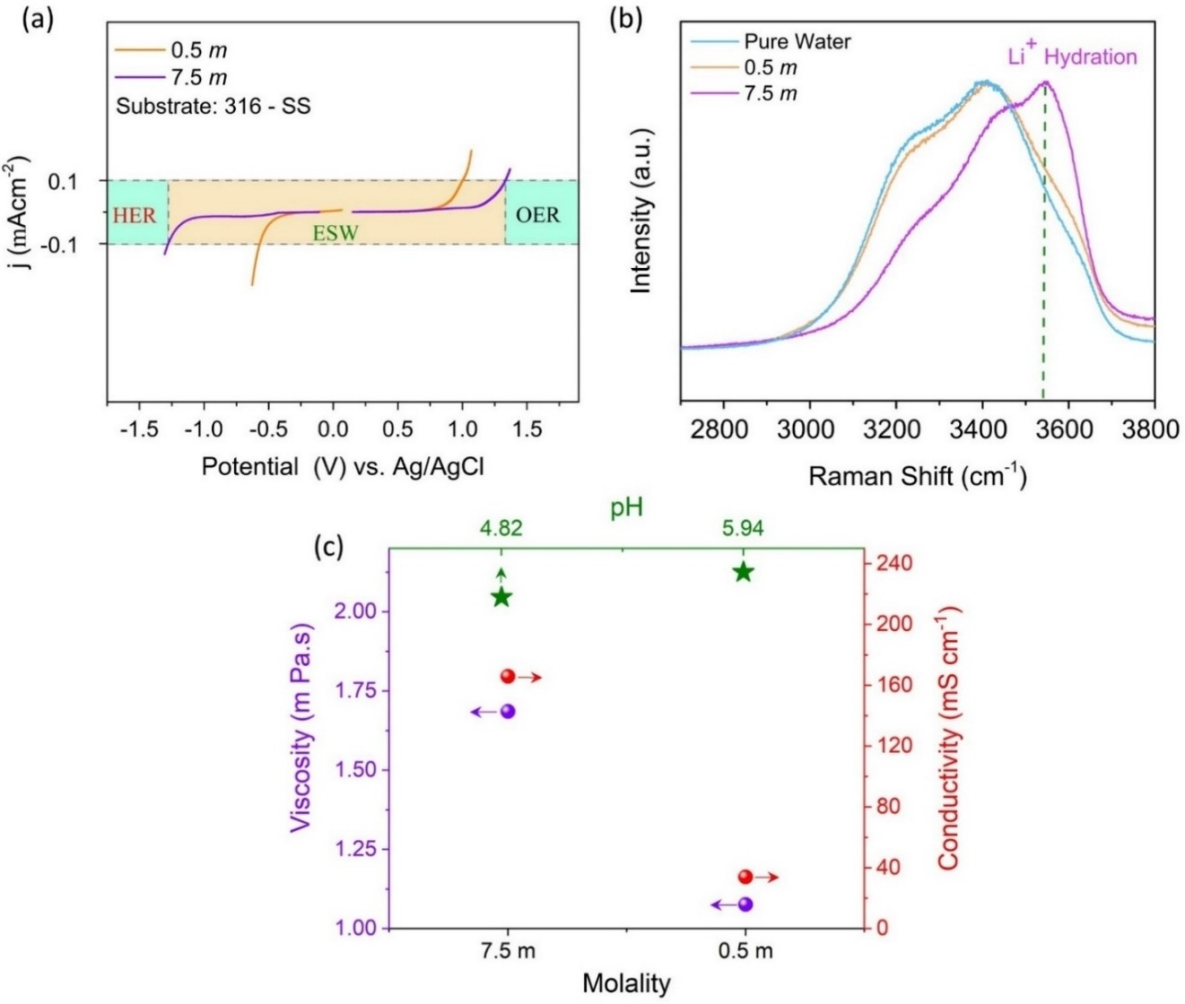
Electrochemical stability window (ESW) test of 7.5 *m* LiClO_4_ WiSE vs 0.5 *m* LiClO_4_ on 316‐stainless steel substrate (a), Raman spectra in the wavenumber region corresponding to the OH‐stretching vibrations of 7.5 m WiSE, diluted electrolyte, and pure water (b), and ionic‐conductivity, pH and viscosity of 7.5 *m* LiClO_4_ WiSE vs 0.5 *m* LiClO_4_ (c).

Electrochemical stability window (ESW) of as‐formulated 7.5 *m* LiClO_4_ ‐WiSE was determined in comparison to low‐concentration 0.5 *m* aqueous electrolyte of the same salt by linear sweep voltammetry (LSV) on a stainless steel (type‐316) electrode vs an Ag/AgCl reference electrode. Figure 1 (a) presents the LSV results where 7.5 *m* electrolyte demonstrates both high oxidative and reductive windows with an overall 2.8 V of ESW. However, 0.5 *m* demonstrated a much narrower window of ~1.7 V, which can be linked with excess free H‐bound water in the dilute electrolyte compared to the more ion‐bound water in the WiSE.^[32]^ This characteristic is evidenced in the Raman spectra of the OH‐stretching region in Figure 1 (b). Pure water and the 0.5 *m* electrolyte have a similar spectrum with the typical broadband peak containing both the O−H symmetric (~3250 cm^−1^) and asymmetric (~3400 cm−1) stretching vibration modes of water molecules due to diverse hydrogen‐bonding environments among water molecules.^[33]^ However, at higher salt concentrations peak narrowing occurs, particularly in “water‐in‐salt” systems, the strong ions coordination results in a more ordered solvation structure. For the case of 7.5 m LiClO_4_ WiSE, the broad O−H stretching vibration bands diminish, leading to the emergence of a sharp peak at 3530 cm^−1^. This peak is indicative of water molecules predominantly participating in the solvation sheath of cations, characterized by minimal hydrogen bonding interactions. In other words, corresponds to cation‐solvated water molecules with negligible hydrogen bonding. This transformation reflects a shift toward a crystalline hydrate‐like environment, where the hydration of Li^+^ is notably enhanced due to the high molar ratios of Li^+^ to water therein.^[34]^ A similar phenomenon demonstrating the absence of the free‐water molecules, and the widening of the stability window has also been observed in our previous studies of other metal perchlorate WiS‐electrolyte systems[^13^, ^35^, ^38^]

Figure 1(c) presents other physical characteristics of the 7.5 m WiSE in comparison to the 0.5 *m* electrolyte. The WiSE was found to be mildly acidic with a pH value of 4.82 vs. 5.94 for 0.5 *m* electrolyte and demonstrated high ionic conductivity of 160.9 mS cm^−1^, where only 32.9 mS cm^−1^ was observed for the dilute electrolyte. However, the viscosity of the 7.5 *m* WiS electrolyte was found to be comparatively higher than that of the dilute electrolyte. Symmetric SCs based on activated carbon (AC‐YEC‐8 A) were assembled using the 0.5 *m* dilute (0.5‐SC) and 7.5 *m* concentrated electrolytes (7.5‐SC) (see details in the experimental section).

The morphology, BET surface area, and pore‐volume distribution of the pristine activated carbon are presented in Figure S1(a–c) (SI information). The BET surface area of AC‐YEC‐8 A carbonaceous material has been calculated using Rouquerol's criteria.^[39]^ A high BET surface area of 2099 m^2^ g^−1^ was found for the pristine AC‐YEC‐8 A sample with high nitrogen adsorption for low relative pressures and a plateau for higher relative pressures. The AC‐YEC‐8 A material exhibits an N_2_ physisorption (adsorption‐desorption) isotherm of type I according to the IUPAC classification, indicating mainly micropores in the structure (pores lower than 2 nm).^[40]^ Furthermore, the pore size distribution was determined using the Density‐Functional‐Theory (DFT) method (for slit pores). It further confirms that the sample contains mainly pores in the range of micropores (<2 nm).^[41]^

**Figure 2:**
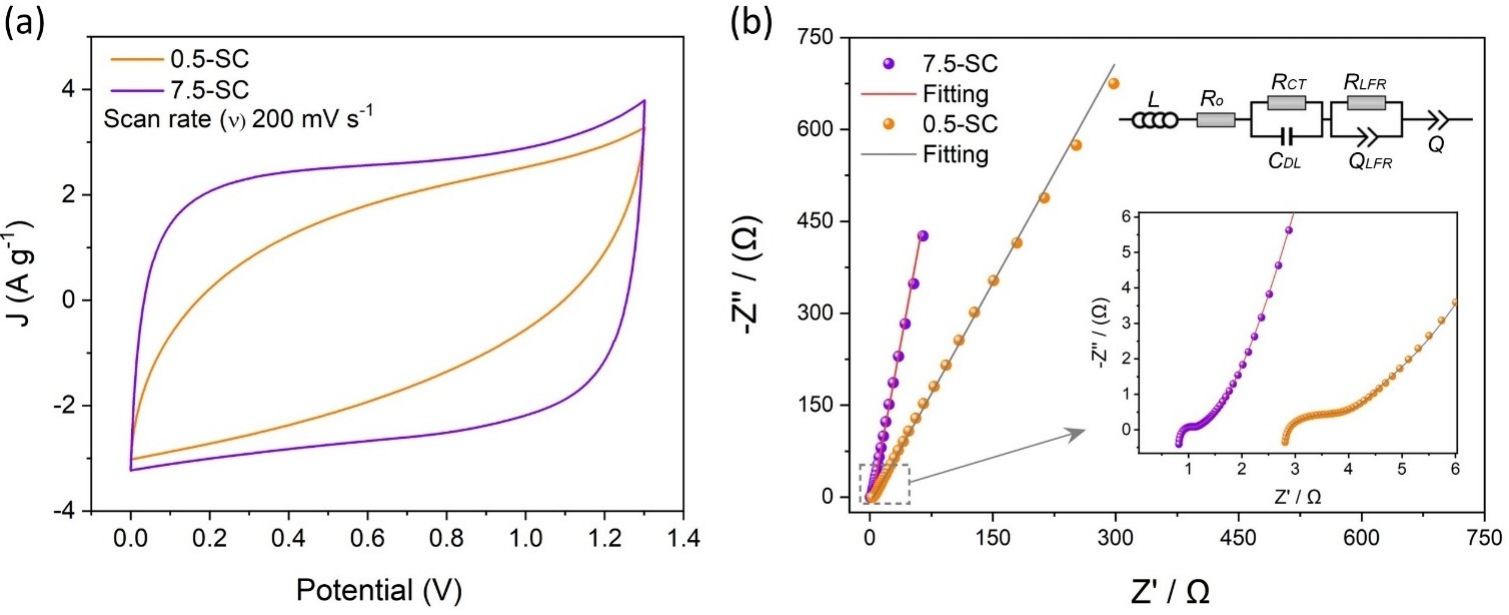
CV of 7.5‐SC and 0.5‐SC at 200 mV s^−1^ (a), and Nyquist plots of both systems in the frequency range of 0.1 Hz–100 kHz in the frequency range of 0.1 Hz to 100 kHz (b).

Figure 2(a) shows the CV of both 7.5‐SC and 0.5‐SC at the scan rate of 200 mV s^−1^. 7.5‐SC with WiSE has a more rectangular CV shape with higher gravimetric current values than the 0.5‐SC. The result shows that the SC based on the highly concentrated aqueous electrolyte has a higher capacitance than the SC based on the diluted electrolyte. The higher capacitance of 7.5‐SC can be assigned to the high concentration of the ionic species in 7.5 *m* WiSE and wide stability window. Furthermore, the WiSE‐based system offers lower charge transfer and ion‐diffusion resistance, as presented in Nyquist plots in Figure 2 (b) and corresponding equivalent circuit. The fitted values for the circuit elements are presented in Table S1. Further, in contract to the dilute electrolytes the increased ionic strength in the concentrated electrolyte shortens the Debye length, improving ion screening and charge transfer at the electrode‐electrolyte interface, which significantly enhances the electrochemical performance and stability of the supercapacitor.^[42]^

**Figure 3:**
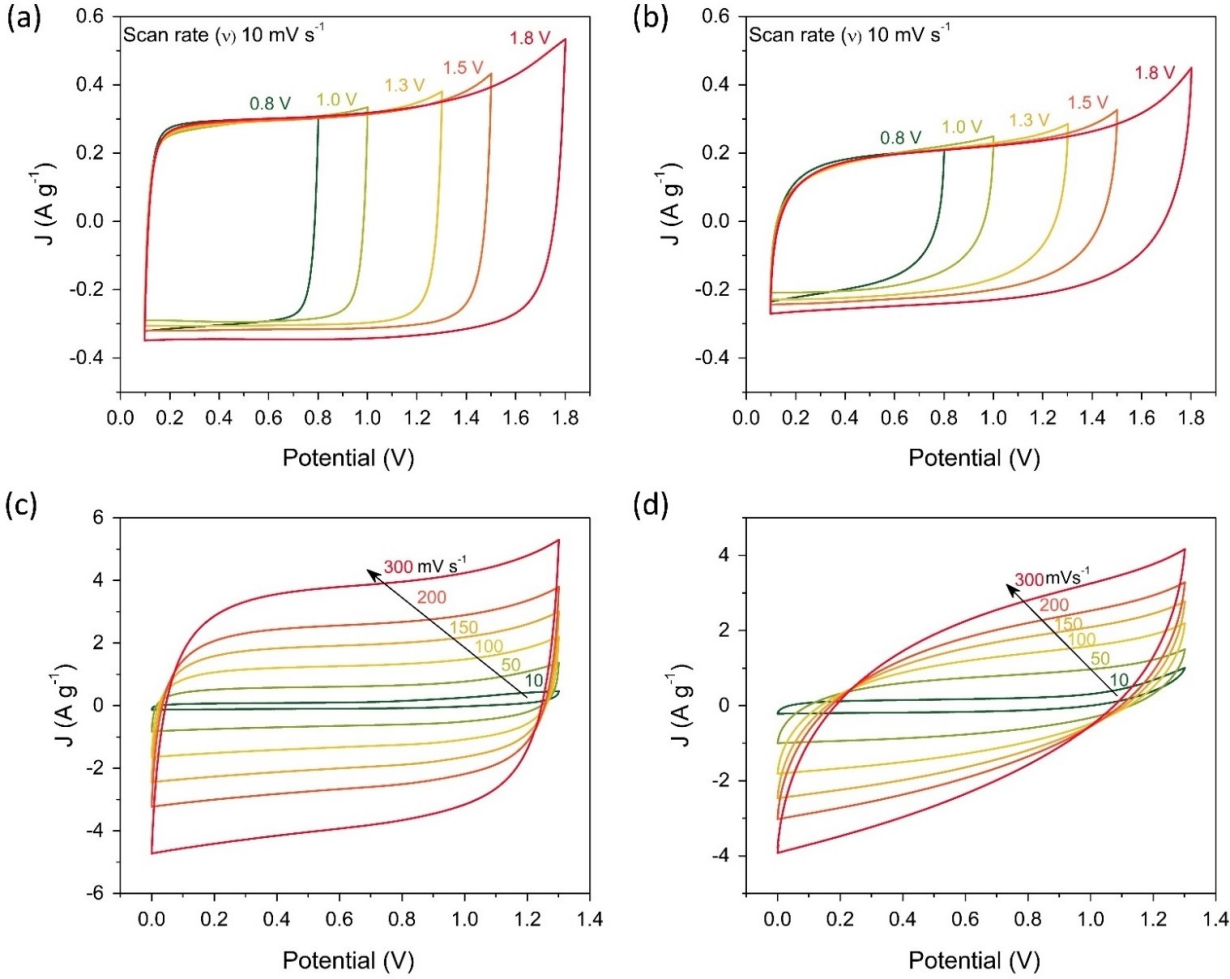
Voltage window determination for cycling and CV at various (a) & (b) cut‐off voltages (0.8–1.8 V) and (c) & (d) scan rates (10 to 300 mV s^−1^) for 7.5‐SC and 0.5‐SC, respectively.

In Figure 3(a–b), CVs at different cut‐off voltages were performed at 10 mV s^−1^ for 7.5‐SC and 0.5‐SC. It can be seen that 7.5‐SC performs better for all studied cut‐off voltages of 0.8–1.8 V, demonstrating a more rectangular shape than 0.5‐SC. As the Coulombic efficiency was low at high cut‐off voltages for both SCs, hence the optimum voltage range of 0–1.3 V was selected because it provided 100 % Coulombic efficiency and a high capa‐citance ~38 F g^−1^ for 7.5‐SC, and ~22 F g^−1^ for 0.5‐SC. Further electrochemical performance tests were performed in the voltage range of 0–1.3 V.

Figure 3(c–d) presents CVs for both SCs at different scan rates from 50 to 300 V s^−1^. 7.5‐SC based on WiSE maintained well rectangular‐shaped CVs even at higher scan rates, which was not the case for 0.5‐SC with the dilute electrolyte. Further, 7.5‐SC could be operated at higher current densities, which suggests that the WiSE system could overcome the detrimental effects of concentration polarization and electrolyte decomposition in comparison to the dilute electrolytes.^[41]^ This result demonstrates that the high‐concentrating WISE systems are suitable for the construction of high‐rate performance aqueous SCs. However, higher concentration makes the system expensive and increases the viscosity due to high utilization of salts. Therefore, to address the viscosity challenge of “Water‐in‐salt” electrolytes, the use of small amounts of organic cosolvents or viscosity‐reducing additives (such as trimethyl phosphate or ethylene carbonate, or 1,4‐dioxane (DX)) could improve ion mobility while maintaining the ESW.[^26^, ^44^]

**Figure 4:**
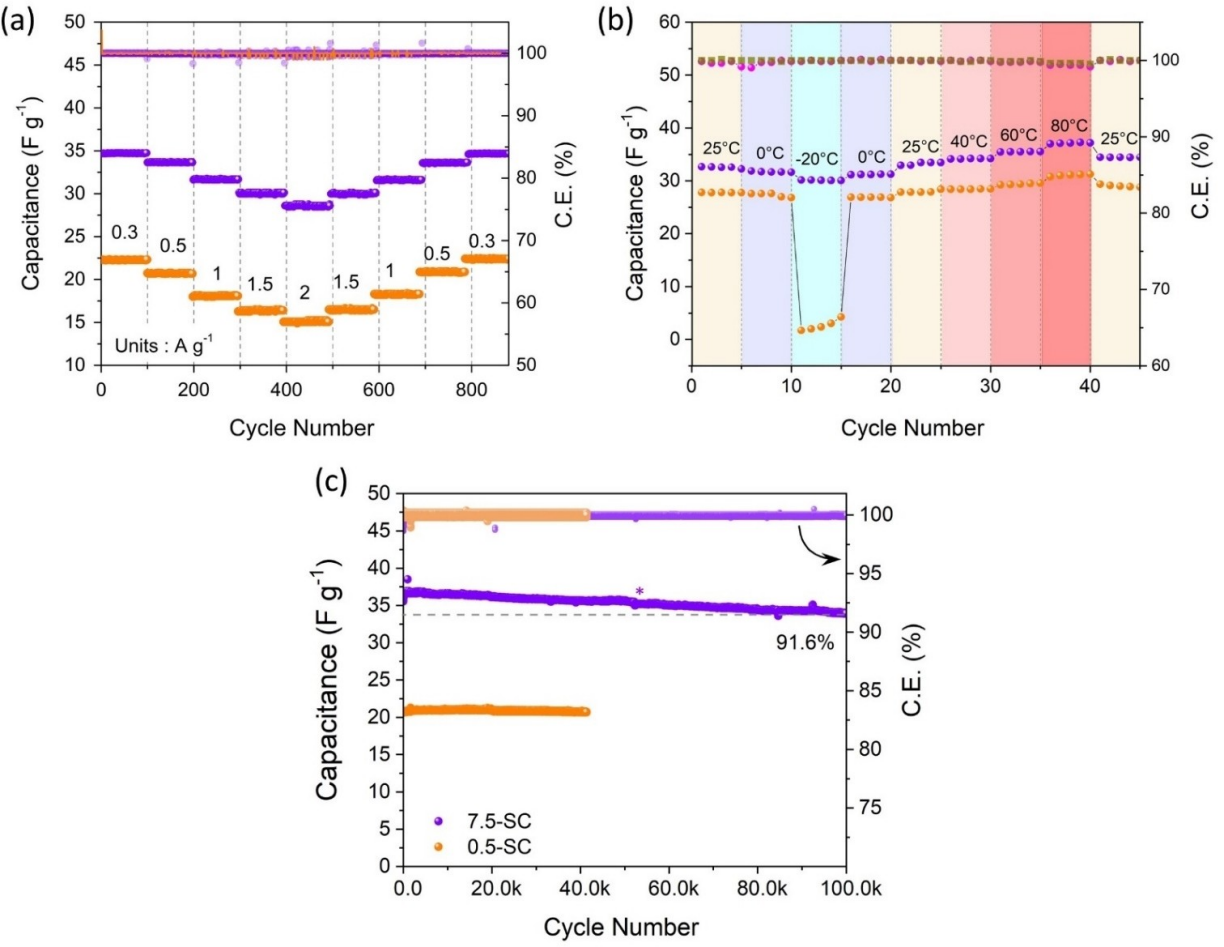
Electrochemical performance test of 7.5‐SC and 0.5‐SC: (a) Rate capability test, (b) cycling test, and (c) wide‐temperature performance test at 0.5 A g^−1^, (* program restarted after ~24 h of rest).

The rate capability performance of SCs is present in Figure 4 (a), where the SCs were galvanostatically charged‐discharged from 300 mA g^−1^ to 2000 mA g^−1^. The Coulombic efficiency for both SCs was found ~100 % at all rates, but 7.5‐SC outperformed 0.5‐SC in terms of capacitance, exhibiting a higher capacitance at all current densities. The use of a highly concentrated 7.5 *m* WISE enhances the ionic strength, which plays a critical role in reducing the Debye length. In general, shorter Debye length in electrolytes results in stronger screening of electrostatic interactions and promotes faster ion movement and charge transfer at the electrode‐electrolyte interface. ^[42]^ These factors, together with the high ionic conductivity and charge carrier concentration, are directly responsible for the superior rate performance observed in the 7.5‐SC. The SC‐7.5 has demonstrated a power density of over 1.3 KW kg^−1^ and an energy density of 8.5 Wh kg^−1^.

An important feature of the present work is the performance of 7.5‐SC in the wide temperature range of −20 °C to 80 °C. Figure 4(b) presents the wide‐temperature range cycling data for 7.5‐SC and 0.5‐SC at 0.5 A g^−1^. The WiSE‐based SC performed well at all over the studied temperature range, even at extreme temperatures, without compromising the capacitance. This was not the case for the SC with the dilute electrolyte, whose capacitance dropped significantly at low temperatures below 0 °C. The diluted electrolyte froze and lost ionic conductivity at −20 °C.

Figure S2(a–b) shows a visual comparison of 0.5 m and 7.5 m LiClO₄ electrolytes before and after storage at −20 °C and 80 °C. Unlike the 0.5 m electrolyte, the 7.5 m solution remained mostly liquid at −20 °C (with some salt crystallization) and exhibited significantly lower mass loss at 80 °C. The high concentration of salts in the WiSE suppressed the freezing of water at this cold temperature. Our results show that a high concentration of salts in aqueous electrolytes can effectively suppress water freezing by enlarging the transformation energy gap between water and ice by strongly binding water molecules to ions and distorting H‐bonding in the WiSE, which results in the high‐ionic conductivity and liquid electrolyte character of WiSE at subzero temperatures Another important feature of the reported SC with LiClO_4_ ‐based WiSE is its fantastic stability and long cycle life, as shown in Figure 4(c), where 7.5‐SC demonstrated ultra‐long cycling of over 100 000 cycles with a capacity retention of over 91 %. 0.5‐SC has also shown good stability and capacity retention, but with a much lower capacitance value and cycle life. This is attributed mainly to the lower number of charge carriers, higher polarization and progressive electrolyte decomposition due to limited ESW of the dilute electrolyte.

## Conclusions

In conclusion, our study demonstrated the successful application of a Li‐perchlorate‐based “water‐in‐salt” electrolyte (WiSE) in symmetric supercapacitors using activated carbon (AC) as the electrode material. The supercapacitor based on the LiClO_4_‐based WiSE demonstrated exceptional stability and a remarkable long‐cycle life of over 100,000 cycles at 500 mA g^−1^. Moreover, the supercapacitor exhibited reliable performance across a wide temperature range (−20 °C to 80 °C), showcasing the potential of LiClO_4_‐based WiS electrolytes for all‐season applications without compromising power densities and other performance metrics. Our findings highlight the benefits of highly concentrated aqueous electrolytes and contribute to the advancement of supercapacitors by expanding the operating potential windows and working‐temperature ranges of aqueous supercapacitors. Future research should focus on optimizing the LiClO_4_‐based WiSE concentration and exploring its performance with different supercapacitor configurations and electrode materials, especially asymmetric SCs to enhance the operating voltage and capacitance.

## Experimental Section

**Materials**: LiClO_4_.3H2O (Alfa‐Aesar GmbH, Germany), activated carbon AC‐YEC‐8 A (Xiamen Tob New Energy Technology Co., Ltd. China), N‐methyl‐2‐pyrrolidone (NMP) (ROTH®, Germany), conductive carbon‐Super P (Imerys, France), polyvinylidene fluoride (PVDF) (Kynar® HSV 1800, Arkema, France), polytetrafluoroethylene (PTFE)‐treated hydrophobic carbon cloth (Fuel Cell Store, USA) and Whatman® GF/D glass microfiber filters (Mar‐Con, s.r.o., Czech Republic.) were purchased from the respective producers and suppliers and used as received without any treatment or purification.

**Electrolyte Preparation**: The supersaturated 7.5 *m* (mol/kg) or water‐in‐salt electrolyte (WiSE) and dilute 0.5 *m* aqueous electrolytes were prepared by mixing the respective quantities of LiClO_4_.3H_2_O and deionized water (conductivity <0.26 μScm^−1^) under continuous stirring.

**Electrode Preparation and Supercapacitor Fabrication**: Electrodes were prepared by mixing AC‐YEC‐8 A, super‐P and PVDF binder dissolved in the NMP disperser (25 mg ml−^1^) in the mass ratio of 8 : 1 : 1 by a pestle in a mortar to get a homogenous slurry. The as‐obtained slurry was pasted on a PTFE‐treated carbon cloth substrate (Fuel Cell Store, USA). The as‐pasted cathode film was dried under vacuum at 80 °C overnight prior to being cut into 8 mm dia electrodes with mass loading of cca. 2 mg cm^−2^. Symmetrical supercapacitors were fabricated in 2032‐coin cells by using as‐prepared electrolytes (7.5 *m* and 0.5 *m*) ~80 μl, AC‐YEC‐8 A electrodes with a single layer of a GF/D glass microfiber separator for the electrochemical measurements.

**Electrochemical Performance Measurements**: The electrochemical stability window of the freshly prepared electrolytes was assessed on a 316‐stainless steel electrode (W.E.) in a standard 3‐electrode set‐up, where platinum (Pt) was used as a counter electrode (C.E.) and leak‐free Ag/AgCl‐reference electrode (R.E.). The potentiostatic cyclic voltammetry (CV), linear sweep voltammetry (LSV), and electrochemical impedance spectroscopy (EIS) were performed by using a potentio/galvanostat (Metrohm Autolab PGSTAT302 N) on the as‐assembled 2‐electrode type cells. The galvanostatic charge/discharge (GCD) and cycle life tests were carried out using a battery tester (Neware, China) at constant (dis)charging current densities at room temperature in 2‐electrode‐type cells. *All the electrochemical measurements were done on the base of the total active mass of both electrodes. In‐operando* wide‐temperature electrochemical performance tests were done by placing the coin cell inside the Linkam HFS350EV‐PB4 Heating Stage (temperature range −195° to 350 °C) and controlling the temperature and galvanostatic charge‐discharge by a computer program.

**Characterization**: A TESCAN scanning electron microscope (SEM) was used for the analysis of the surface morphology of the electrode. The pH and ionic conductivity of the electrolytes were measured using a Metrohm pX1000 module integrated with Autolab PGSTAT302 N and WTW® portable Cond 3310 SET, respectively. The (de)adsorption of nitrogen was carried out with the ASAP 2020 apparatus (Micromeritics) at 77 K. The sample was degassed before analysis at 250 °C in a vacuum for 3 h. The surface area was determined by the BET (Brunauer, Emmett, Teller) equation. The pore size distribution was determined using the Density‐Functional‐Theory (DFT) method (for slit pores). Raman spectroscopy was performed on a Renishaw Invia™ confocal Raman spectroscope equipped with an optical microscope, a He−Cd blue laser (442 nm excitation wavelength), and 2400 l/mm diffraction grating. All data was processed using Origin (OriginLab Corp.). EIS data were evaluated by means of the RelaxIS 3® software suite (rhd instruments GmbH & Co. KG).

**Calculation of specific capacitance, specific energy, and specific power density of supercapacitor**: The specific capacitance C (F g^−1^) of this device was calculated from GCD by following equation:

$$C=\frac{I\cdot \Delta t}{m\cdot \Delta U}\;$$

where I (A) is the loaded current, Δt (s) is the discharge time, m (g) is the mass of AC electrodes (both) inside the device, and U (V) is an operating voltage. The specific energy density E (Wh kg^−1^) was calculated using the equation below:

$$E=\frac{C\cdot U^{2}}{7.2}\;$$

where C (F g^−1^) is the specific capacitance, and U (V) is the operating voltage of the device. The specific power density P (W kg^−1^) was calculated by the following equation:

$$P=\frac{E\cdot 3600}{\Delta t}\;$$

where E (Wh kg^−1^) is energy density, and Δt (s) is the discharge time.

## Conflict of Interests

The authors declare no conflict of interest.

1

## Supporting information

As a service to our authors and readers, this journal provides supporting information supplied by the authors. Such materials are peer reviewed and may be re‐organized for online delivery, but are not copy‐edited or typeset. Technical support issues arising from supporting information (other than missing files) should be addressed to the authors.

Supporting Information

## Data Availability

The data that support the findings of this study are openly available in zenodo.org at https://doi.org/10.5281/zenodo.13132695, reference number 13132695.
